# Screening for anxiety and depression: reassessing the utility of the Zung scales

**DOI:** 10.1186/s12888-017-1489-6

**Published:** 2017-09-08

**Authors:** Debra A. Dunstan, Ned Scott, Anna K. Todd

**Affiliations:** 1 School of Behavioural, Cognitive and Social Sciences, University of New England, Armidale, NSW 2351 Australia; 2 School of Behavioural, Cognitive and Social Sciences, University of New England, Armidale, NSW 2351 Australia

**Keywords:** Depression/anxiety screening, Depression anxiety stress scale (DASS), Zung self-rating depression scales (SDS), Zung self-rating anxiety scales (SAS)

## Abstract

**Background:**

While the gold standard for the diagnosis of mental disorders remains the structured clinical interview, self-report measures continue to play an important role in screening and measuring progress, as well as being frequently employed in research studies. Two widely-used self-report measures in the area of depression and anxiety are Zung’s Self-Rating Depression Scale (SDS) and Self Rating Anxiety Scale (SAS). However, considerable confusion exists in their application, with clinical cut-offs often applied incorrectly. This study re-examines the credentials of the Zung scales by comparing them with the Depression Anxiety Stress Scale (DASS) in terms of their ability to predict clinical diagnoses of anxiety and depression made using the Patient Health Questionnaire (PHQ).

**Method:**

A total sample of 376 adults, of whom 87 reported being in receipt of psychological treatment, completed the two-page version of the PHQ relating to depression and anxiety, together with the SDS, the SAS and the DASS.

**Results:**

Overall, although the respective DASS scales emerged as marginally stronger predictors of PHQ diagnoses of anxiety and depression, the Zung indices performed more than acceptably in comparison. The DASS also had an advantage in discriminative ability. Using the current recommended cut-offs for all scales, the DASS has the edge on specificity, while the Zung scales are superior in terms of sensitivity. There are grounds to consider making the Zung cut-offs more conservative, and doing this would produce comparable numbers of ‘Misses’ and ‘False Positives’ to those obtained with the DASS.

**Conclusions:**

Given these promising results, further research is justified to assess the Zung scales ability against full clinical diagnoses and to further explore optimum cut-off levels.

## Background

The gold standard for making diagnoses based on the Diagnostic and Statistical Manual of Mental Disorders (DSM; [1]) is the copyrighted Structured Clinical Interview for DSM Disorders (SCID; [2]). However, this is a lengthy instrument to administer and hence researchers and clinicians often screen for the presence and severity of both disorders using self-report psychometric tools developed for this purpose. Self-report measures of mental disorders may be criterion-referenced or norm-referenced. Criterion-referenced measures provide a provisional diagnosis based on the endorsement of criteria listed in published diagnostic classification systems. Individuals are diagnosed with or without a disorder based upon the presence or absence of these criteria [3, 4]. In contrast to criterion-referenced measures, norm-referenced measures compare individuals’ test results to those of an appropriate peer or normative group. These scales typically suggest score ranges linked to symptom severity descriptors, and have a “clinically significant” total scale score cut-off point beyond which scores are considered indicative of the presence of a disorder. Moreover these measures are often used to measure therapeutic progress/outcomes as well as being frequently employed in research studies.

Anxiety and depression are the most prevalent of mental illnesses contributing to the global disability burden [5, 6]. Numerous norm-referenced scales have been created for the measurement of anxiety and depression. Research has shown, however, that many of these measures have poor discriminative ability [7, 8] and, rather than being specific to anxiety or depression, tap only a general ‘emotionality’ [9, 10].

The Self Rating Depression Scale [SDS; 11] and the Self-Rating Anxiety Scale [SAS; 12] are two such norm-referenced scales. Both are 20 item Likert scales, in which items tap psychological and physiological symptoms and are rated by respondents according to how each applied to them within the past week, using a 4-point scale ranging from 1 (*none, or a little of the time*) to 4 (*most, or all of the time*). The choice of SDS items was based on factor analytic studies of depression symptoms [11], whereas the SAS taps affective symptoms based on diagnostic criteria listed in the major American psychiatry literature [12]. These scales continue to be widely utilised but a recent review of the literature revealed that considerable confusion exists in their application, with clinical cut-offs frequently being incorrectly applied [13]. This confusion stems from the fact that Zung chose to convert raw scores (range 20–80) into index scores (range 25–100) with clinical cut-offs being given in terms of the latter. However, these cut-offs have frequently been applied to raw scores, producing a substantial elevation in the degree of symptom severity required to meet clinical significance. Problems also exist with the application of severity ranges [13].

In the light of this research, and the ongoing substantial usage of both the Zung scales, this study seeks to re-examine these scales’ credentials as screeners for depression and anxiety. It does so by comparing these scales with one of the most prominent and respected norm-referenced scales of this kind: the Depression Anxiety Stress Scale [DASS; 14]. The DASS has sub-scales that differentiate anxiety, depression, and a third factor, labelled stress. In developing the scale, the authors, Lovibond and Lovibond [14], focused on including only aspects unique to each disorder, which led to the omission of somatic items (e.g., sleep disturbance, loss of libido, fatigue, appetite change) as well as items related to suicidal ideation, which failed to correlate with the depression subscale. The scale’s three-factor solution has been replicated and the scale has been reported to reliably distinguish samples of patients with anxiety or depression from non-clinical participants [15, 16]. It is also reported to be an improvement on the older measures of anxiety and depression, for example, showing greater separation in factor loading than the Beck Depression and Anxiety Inventories [14].

The current study examines the ability of the Zung and DASS scales to predict clinical diagnoses made using the Patient Health Questionnaire (PHQ; [17]). The PHQ was developed from the Primary Care Evaluation of Mental Disorders (PRIME-MD) interview, which was the first instrument designed for use in primary care to diagnose disorders based on DSM criteria. The PHQ is commonly employed in both clinical practice and research, and has been found to be a reliable and valid diagnostic tool [18]. Hence, while the structured clinical interview remains the gold standard for diagnosis, it is argued that the PHQ is a suitable benchmark against which to compare these norm-referenced scales.

### The current study

In keeping with the claims made by Lovibond and Lovibond [14], it was hypothesised that the DASS subscales would emerge as stronger predictors of - and offer greater discrimination between - the two classes of disorder. In the light of the differences in item selection between types of measures, it was also hypothesised that there would be a mismatch in agreement between the different measures. However, as this was exploratory research, no estimation was made about the size or nature of these discrepancies. Rather the research sought to explore differences between the DASS and Zung measures in terms of the balance between sensitivity and specificity.

## Method

### Participants

In accordance with the requirement to study diagnosis in the entire population [19, 20], a total sample of 376 participants were recruited from community and clinical populations. Of these, 340 were undergraduate psychology students who participated in return for one Research Participation Opportunity point upon completion. The remaining 36 participants were clients presenting for treatment at psychological services in the New England area of New South Wales, Australia, who volunteered in response to publicity fliers provided by their psychologist. Psychologists were not paid for their assistance in the study but were thanked with a small gift valued at less than $10. In total 87 participants (21 men and 66 women), with a mean age of 39.53 (range 18 years to 74 years), who reported receiving treatment for psychological issues from either a medical practitioner or psychologist were classified as members of the clinical sample. The non-clinical sample consisted of 289 participants (54 males, 235 females) with a mean age of 31.28 (range 18 years to 64 years). Participants under 18 were excluded because the psychometric measures utilised in the study were designed for adults only.

### Procedure

Undergraduate psychology students completed the study online. Clients of psychological services used pencil and paper measures. In addition to the collection of demographic and medical information, the SAS, the SDS, the DASS and the two-page version of the PHQ, covering only depressive and anxiety disorders, were all administered. The order of presentation of these four measures was randomised and all information collected at a single sitting.

### Measures used

As detailed above, both the SAS and the SDS are 20 item Likert scales, with raw scores that range from 20 to 80, which are converted to index scores by dividing the sum of the raw scores by 80, and multiplying by 100. A raw score - index score conversion table is provided by Zung [12, p. 376].

SAS items tap both affective and somatic symptoms; 15 express a negative experience such as “I feel afraid for no reason at all” and 5 express a positive experience and are reverse scored, such as “I can breathe in and out easily”. Zung [21] noted in an early study that all “normal subjects” returned an SAS Index score below 50, but later he set an Index score of 45 (raw score = 36) as a cut-off point for clinically significant anxiety [22]. While higher scores indicate greater severity of anxiety symptomology, score ranges for degrees of severity have not been published in the scientific literature. The SAS has been shown to have good internal consistency with a Cronbach’s alpha of .82 [10]; fair concurrent validity, correlating significantly (.30) with the Taylor Manifest Anxiety Scale [12]; and to distinguish both between clinical and non-clinical groups and between patients diagnosed with anxiety disorders and those with other psychiatric diagnoses [12].

SDS items also tap both affective and somatic symptoms; 10 express negative experience such as “I feel down-hearted and blue” and 10 express positive experience and are reverse scored such as “I eat as much as I used to”. An SDS Index score of 50 (raw score = 40) suggests clinically significant symptoms with the following three levels of severity ratings: Index scores 25–49 (raw scores 20–40) *Normal*; 50–59 (raw scores 41–47) *Mild to Moderate*; 60–69 (raw scores 48–55) *Moderate to Severe*; and 70 and over (raw scores 56 and over) *Severe* [23]. The SDS has fair internal consistency, with a split-half reliability of .73. An alpha coefficient of .68 was reported by Deforge and Sobal [24], while other authors have reported .79 [25] and .81 [10]. Reported correlations with other depression scales include .41 with the Hamilton Rating Scale [26], .54 with the Depression Adjective Checklist, and .68 with the Beck Depression Inventory [10].

The Depression Anxiety Stress Scale (DASS) developed by Lovibond and Lovibond is a 42 item self-report measure of anxiety, depression and stress. Depression items include ‘“I felt down-hearted and blue” and “I felt I had nothing I could look forward to”; Anxiety items include “I felt I was close to panic” and “I experienced trembling (e.g., in the hands)”; and Stress items include “I found it hard to wind down” and “I was intolerant of anything that kept me from getting on with what I was doing”. Based on their experience over the previous four weeks, respondents rate the items on a 4-point scale ranging from 0 (*did not apply to me at all*) to 3 (*applied to me very much, or most of the time*). Items in each subscale are summed to provide scores for symptoms of depression, anxiety and stress, with higher scores indicating greater severity of symptomatology described within three levels. Score ranges for severity ratings for the depression subscale are: 0–9 (*Normal),* 10–13 (*Mild*), 14–20 (*Moderate*), 21–27 (*Severe*) and 28+ (*Extremely Severe)*. Ranges and ratings for the anxiety subscale are: 0–7 (*Normal)*, 8–9 (*Mild*), 10–14 (*Moderate*), 15–19 (*Severe*) and 20+ (*Extremely Severe);* and for the stress subscale are: 0–14 (*Normal*), 15–18 (*Mild*), 19–25 (*Moderate*), 26–33 (*Severe*) and 34+ (*Extremely Severe*). The scale has demonstrated reliability, with alpha values of .91 for the depression subscale, .84 for the anxiety subscale, and .90 for the stress subscale. The scale has also been shown to have concurrent validity with correlations of .74 between the depression subscale and the Beck Depression Inventory and .81 between the anxiety subscale and the Beck Anxiety Inventory [14].

The PHQ is a brief, user-friendly self-report measure with items corresponding to a range of diagnostic criteria taken from the fourth edition (revised) of the DSM. As detailed above, this study utilised the two-page version, covering Major Depressive Disorder and Other Depressive Disorder (9 items), and Panic Disorder and Other Anxiety Disorder (22 items). Compared with diagnoses made by mental health professionals, the sensitivity of the PHQ is 73% and the specificity 94% [17]. It should be noted that all the criteria on which the PHQ is based remain unchanged in the current edition of the DSM [1, 27].

## Results

### Participants reaching diagnostic criteria on the PHQ

Across the combined sample, 61 participants (34 and 27 from the clinical and non-clinical samples respectively) reached the PHQ diagnostic criteria for some form of depressive disorder and 54 (34 from the clinical sample, 23 from the non-clinical) for some form of anxiety disorder. Of those satisfying the criteria for a depressive disorder, 43 (70%) met the criteria for a Major Depressive Disorder (MDD). Of those satisfying the criteria for an anxiety disorder, 25 (46%) met the criteria for Panic Disorder, and 35 (65%) the criteria for Other Anxiety Disorder. Levels of comorbidity between anxiety and depressive disorders were considerable with 31 of the above participants suffering from both a depressive and an anxiety disorder. These participants were predominantly from the clinical sample (23 compared with only 8 from the non-clinical).

### Internal consistency

Both the Zung scales and the three DASS subscales were found to have acceptable levels of internal consistency. Cronbach’s alphas of .86 and .84 were found for the SDS and SAS, while the comparable figures for the DASS Depression, Anxiety, and Stress subscales were .96, .88 and .94 respectively. Table 1 details means, standard deviations, and severity range descriptors for these measures within both samples.

**Table 1:** Mean scores (S.D.’s) and Severity Range Descriptors for the Clinical and Non-Clinical Sample on the Norm-Referenced Measures

Measure	Total Sample		Non-Clinical Sample		Clinical Sample	
DASS42						
Depression Subscale	7.16 (8.76)	Normal	5.47 (6.83)	Normal	12.77 (11.70)	Mild
Anxiety Subscale	4.94 (5.61)	Normal	3.80 (4.29)	Normal	8.74 (7.55)	Mild
Stress Subscale	10.93 (8.25)	Normal	9.29 (6.57)	Normal	16.34 (10.65)	Mild
SAS Total Raw Score	34.09 (8.18)	Normal	32.54 (7.36)	Normal	39.25 (8.66)	Clinically Significant
SDS Total Raw Score	38.41 (9.43)	Normal	36.67 (8.32)	Normal	44.21 (10.55)	Mild - Moderate

### Comparing the Zung and DASS scales’ predictive ability

A preliminary analysis showed high levels of correlation between all five DASS and Zung scales. Correlations between the three DASS subscales ranged from .70 to .73, while the correlation between the two Zung scales was .80. Similarly high correlations were found between the corresponding DASS and Zung scales: .78 for the DASS Depression subscale with the SDS, and .76 for the DASS Anxiety subscale with the SAS.

Investigation of these scales’ predictive abilities followed two distinct routes. First a series of logistic regressions were conducted exploring the ability of the relevant DASS and Zung scales to correctly predict diagnoses of anxiety/depressive disorders as made from the PHQ. These analyses were, by nature, independent of the prescribed cut-off scores. However, in addition, sensitivity and specificity analyses were conducted using the current recommended cut-offs for each scale.

As far as the logistic regressions were concerned, the combined sample size was adequate [28], there were no problems with multicollinearity, and independence of errors was assumed as the samples did not have shared networks. There were a number of univariate outliers in the total sample: however, all cases were retained because the inclusion of extreme scores did not shift the variable means into the clinical range. A number of multivariate outliers were also identified but further inspection suggested these outliers were valid members of their respective samples and not qualitatively different from other participants. Moreover, excluding these cases had minimal impact on results, hence all participants were retained. Within these analyses, comparisons of the relative strength of individual predictors relied on the method recommended by Kaufman [29]: namely calculating semi-standardised coefficients (*SS* ^*ΔP*^), which reflect the change in outcome probability for a standardised unit increase in the predictor. These coefficients are essentially equivalent to standardized betas in multiple regression.

#### Ability to predict depression

As the DASS and Zung scales are rarely used in tandem, we began by conducting two separate logistic regression analyses, one with the DASS Depression subscale as the predictor and the other with the SDS. Both scales were strong predictors of PHQ depression diagnoses, with *p* < .001 for each analysis. Calculations of *Nagelkerke R* ^*2*^ revealed that the DASS Depression subscale accounted for 58% of the variance in PHQ diagnosis outcome, while the SDS was somewhat weaker, accounting for 49%.

In addition, a further logistic regression was conducted with both the DASS Depression subscale and the Zung SDS, entered as predictors. Interestingly, *Nagelkerke R* ^*2*^ for the combined model is only slightly greater than when the DASS depression subscale is entered alone, indicating that the addition of the SDS does little to extend the amount of variance explained. Both scales remain significant predictors but their respective *SS* ^*ΔP*^ values indicate that, when used alongside each other, the DASS scale makes the greater contribution, a standardised unit increase producing a 20.9% increase in the predicted probability of diagnosis, compared to an 11.6% increase for the SDS (Table 2).

**Table 2:** Results of separate Logistic Regression Analyses assessing the ability of the DASS Depression Subscale and the Zung SDS, to individually and jointly predict depression disorder diagnoses on the PHQ

A. DASS Depression Subscale only (*Nagelkerke R* ^*2*^ = .58)				
	*b*	Sig.	Odds Ratio	*SS* ^*ΔP*^
DASS Depression Scale	.235	<.001	1.264	.286
B. Zung SDS only (*Nagelkerke R* ^*2*^ = .49)				
	*b*	Sig.	Odds Ratio	*SS* ^*ΔP*^
* SDS*	.212	<.001	1.236	.278
C. Both scales entered as predictors (*Nagelkerke R* ^*2*^ = .60)				
	*b*	Sig.	Odds Ratio	*SS* ^*ΔP*^
DASS Depression Scale	.173	<.001	1.189	.209
SDS	.090	.008	1.094	.116

#### Ability to predict anxiety

Similar logistic regression analyses were used to investigate the ability of the respective DASS and Zung scales to predict a diagnosis of anxiety on the PHQ. Results mirrored those for depression. When the scales were entered as solus predictors, both were significant at the *p* < .001 level, with the DASS Anxiety subscale accounting for slightly more variance (47% versus 40%) of the variance in PHQ diagnosis, compared to the SAS. Once again, the amount of variance accounted for was only marginally increased (to 49%) by including both scales as predictors. Moreover, while both scales remain significant predictors, *SS* ^*ΔP*^ values again indicate that the DASS subscale makes a greater contribution, with a standardised unit increase producing a 15.1% increase in the predicted probability of diagnosis, compared to an 8.3% increase for the SAS (Table 3).

**Table 3:** Results of separate Logistic Regression Analyses assessing the ability of the DASS Anxiety Subscale and the Zung SAS, to individually and jointly predict anxiety disorder diagnoses on the PHQ

A. DASS Anxiety Subscale only (*Nagelkerke R* ^*2*^ = .47)				
	*b*	Sig.	Odds Ratio	*SS* ^*ΔP*^
DASS Anxiety Scale	.297	<.001	1.346	.211
B. Zung SAS only (*Nagelkerke R* ^*2*^ = .40)				
	*b*	Sig.	Odds Ratio	*SS* ^*ΔP*^
* SAS*	.196	<.001	1.217	.202
C. Both scales entered as predictors (*Nagelkerke R* ^*2*^ = .49)				
	*b*	Sig.	Odds Ratio	*SS* ^*ΔP*^
DASS Anxiety Scale	.215	<.001	1.240	.151
SAS	.082	.016	1.085	.083

### Comparison of the DASS and Zung scales’ discriminant ability

Further logistic regression analyses were conducted to explore the discriminant ability of the respective DASS and Zung scales: these involved using, first, the three DASS subscales and, second, the SDS and the SAS, as predictors of both depression and anxiety disorders on the PHQ. The DASS subscales displayed a high level of discrimination, with only the appropriate subscales emerging as significant predictors of each type of disorder (Table 4). In contrast, while only the SAS was a significant predictor of an anxiety diagnosis, both Zung scales emerged as significant predictors of a depression diagnosis. The SDS was, however, the stronger, a standardised unit increase producing a 19.0% increase in the predicted probability of diagnosis, compared to a 10.6% increase for the SAS (Table 5).

**Table 4:** Results of separate Logistic Regression analyses assessing the ability of (i) the three DASS Subscales and (ii) the two Zung scales to predict depression disorder diagnoses on the PHQ

A. The DASS subscales (*Nagelkerke R* ^*2*^ = .59)				
	*b*	Sig.	Odds Ratio	*SS* ^*ΔP*^
DASS Depression Scale	.209	<.001	1.232	.253
DASS Anxiety Scale	−.023	.631	0.997	−.026
DASS Stress Scale	.062	.067	1.064	.070
B. The Zung scales (*Nagelkerke R* ^*2*^ = .52)				
	*b*	Sig.	Odds Ratio	*SS* ^*ΔP*^
SDS	.147	<.001	1.158	.190
SAS	.095	.005	1.100	.106

**Table 5:** Results of separate Logistic Regression analyses assessing the ability of (i) the three DASS Subscales and (ii) the two Zung scales to predict anxiety disorder diagnoses on the PHQ

A. The DASS subscales (*Nagelkerke R* ^*2*^ = .49)				
	*b*	Sig.	Odds Ratio	*SS* ^*ΔP*^
DASS Anxiety Scale	.244	<.001	1.276	.172
DASS Depression Scale	.003	.911	1.003	.003
DASS Stress Scale	.050	.124	1.051	.051
B. The Zung scales (*Nagelkerke R* ^*2*^ = .41)				
	*b*	Sig.	Odds Ratio	*SS* ^*ΔP*^
SAS	.167	<.001	1.182	.172
SDS	.033	.256	1.033	.038

### Sensitivity and specificity of the norm-referenced measures

Sensitivity and specificity analyses were conducted for the norm-referenced measures using PHQ criteria for depressive and anxiety disorders. (Note: sensitivity refers to true positives, or the proportion of cases that have the condition and are identified as such; specificity refers to true negatives, or the proportion of cases that don’t have the condition and are correctly identified as such). For the DASS Depression and Anxiety Subscales, analyses used the mild severity levels recommended by Lovibond and Lovibond [14] as the cut-off. For the SDS and SAS, the cut-off points recommended by Zung [12, 22] were used: an index score of 50 and above (raw score 40 and above) for depression, and an index score of 45 and above (raw score 36 and above) for anxiety.

With respect to depressive disorders, the DASS subscale recorded 84% for both sensitivity and specifity. The SDS, on the other hand, had a sensitivity of 93% and specificity of 69%. With respect to anxiety disorders, the DASS subscale had 74% sensitivity and 84% specificity, whereas the SAS registered 89% sensitivity and 69% specificity.

For comparison, calculations were also made using the incorrect cut-off points (raw scores of 50 and above) mistakenly applied in a number of past studies [13]. This reduced the sensitivity of the SDS to 56% (specificity 95%) and that of the SAS to 31% (specificity 98%).

## Discussion

### Comparisons of predictive and discriminative ability

The core objective of this research was to assess the ability of the Zung scales (the SDS and the SAS) to predict diagnoses of depression and anxiety. When employed alongside each other in logistic regression analyses, the respective DASS subscales emerge as making a stronger contribution to accurate prediction of PHQ diagnoses. However, the Zung scales still make a significant contribution. Moreover, when used as solus predictors, as is generally the case in clinical practice, the Zung scales perform in broadly comparable terms, accounting for only slightly less of the variance in PHQ diagnoses and with similar increases in the probability of diagnosis for a standardised unit increase in each scale.

A further issue for investigation concerned the ability of the respective DASS and Zung scales to discriminate between depression and anxiety. Previous research has demonstrated high correlations between these measures and this study was no exception with correlations actually somewhat higher than found previously: .80 between the two Zung indices, compared to the .71 reported by Tanaka-Matsumi and Kameoka [10]; and correlations ranging from .70 to .73 between the DASS subscales, compared to results ranging from .50 to .60 reported by Lovibond and Lovibond [14]. Sizeable correlations are to be expected, given the high degree of comorbidity between depression and anxiety. Just over half of those diagnosed with a depressive disorder on the PHQ also met the criteria for some form of anxiety disorder. This is broadly comparable with the findings of the National Comorbidity Survey in which 58% of Major Depressive Disorder sufferers were found to have an anxiety disorder [30]. Despite these high correlations, the DASS subscales matched their designers’ intentions with the depression and anxiety subscales uniquely predicting these PHQ diagnoses. In contrast, while only the SAS was a significant predictor of anxiety, both Zung scales emerged as significant predictors of a depressive disorder diagnosis.

The fact that the SDS emerges as specific to depression, while high scores on the SAS are predictive of both disorders is somewhat surprising, given it was the presence of somatic items on depression scales that Lovibond and Lovibond [14] identified as one of the primary contributors to traditional scales’ poor discriminative ability. However, it has to be remembered that the PHQ on which these diagnoses are based also contains somatic items (as indeed do the DSM criteria from which the PHQ was developed).

### Comparison of screening results

Calculations of sensitivity and specificity for the different scales can be misleading because they depend on the cut-off scores utilised. Using the cut-offs recommended by the scale authors, the DASS scales have superior specificity but the Zung scales have greater sensitivity. However, further investigation revealed that, by modifying the Zung cut-off scores, a similar balance between sensitivity and specificity to that offered by the DASS subscales could be obtained. Specifically, with the cut-off for a depression diagnosis set at an index score of 55 (raw score 44), the SDS has a sensitivity of 80% and a specificity of 82% (compared to 84% on both measures for the DASS depression subscale). Similarly, with the cut-off for an anxiety diagnosis set at an index score of 50 (raw score 40), the SAS has a sensitivity of 72% and a specificity of 84% (compared to 74% and 84% respectively for the DASS anxiety subscale).

**Fig. 1:**
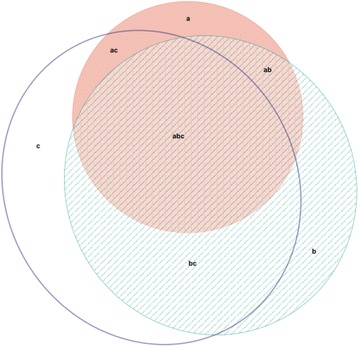
Venn diagram illustrating overlap between diagnoses of depressive disorder on the PHQ (area a – pink ellipse), and positive screens on the DASS depression subscale (area b –green shaded ellipse) and the SDS with a revised cut-off: index scores of 55 and above (area c – clear ellipse). Figure produced using the eulerAPE software developed by Micallef and Rodgers [31]

**Fig. 2:**
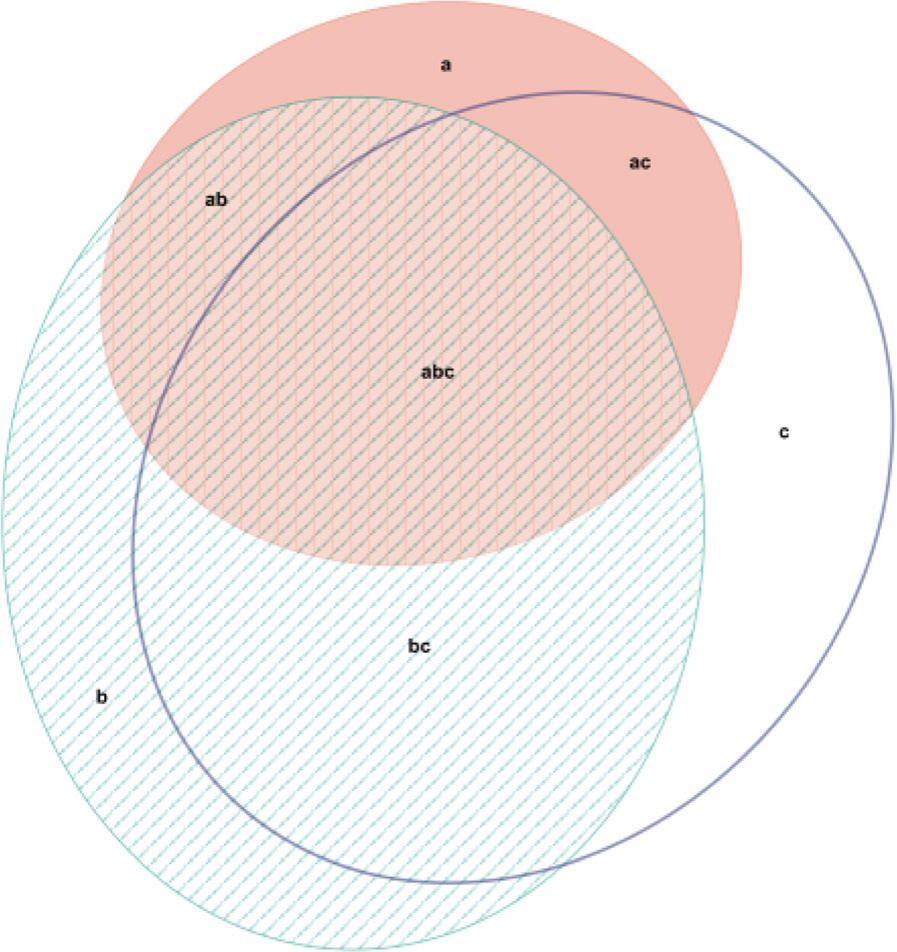
Venn diagram illustrating overlap between diagnoses of an anxiety disorder on the PHQ (area a – pink ellipse), and positive screens on the DASS anxiety subscale (area b –green shaded ellipse) and the SAS with a revised cut-off: index scores of 50 and above (area c – clear ellipse). Figure produced using the eulerAPE software developed by Micallef and Rodgers [31]

These results can also be expressed in terms of the numbers of “Misses” (diagnoses on the PHQ which are not picked up by the DASS and/or Zung scales) and “False Positives” (screen positive on the DASS and/or Zung scale but are not diagnosed on the PHQ) that occur with each scale. Using the revised cut-offs for the Zung scales suggested above, the results for depression are as follows: 12 misses and 58 false positives on the SDS, 10 misses and 49 false positives on the DASS depression subscale. For anxiety there are 15 misses and 50 false positives on the SAS, and 14 misses and 50 false positives on the DASS anxiety subscale. Figures 1 and 2 detail the relative overlap between positive diagnoses on the PHQ and positive screens on the DASS and Zung measures using these revised cut offs.

### Errors in past usage of the Zung scales

Although, on the basis of the above, a case can be made for increasing the cut-off point of the SDS and SAS, it should be noted that the increase involved when raw scores are mistakenly used instead of index scores goes far beyond what is desirable, with sensitivity falling to 56% for the SDS and 31% for the SAS. This means that almost half of those who would qualify for a depressive disorder diagnosis on the PHQ and over two-thirds of those who would qualify for an anxiety disorder diagnosis are likely to have been excluded from the clinical samples in such studies. It is clearly important that such errors be eliminated in any future use of these scales.

## Conclusions

Based on the above results, the Zung scales appear to offer a good alternative to the DASS as screeners for anxiety and depression. It should, however, be noted that the diagnostic measure against which these scales have been assessed is not the gold standard SCID but rather the more economic and less reliable PHQ, a measure which itself produces “Misses” and “False Positives”. These results, however, provide justification for further research to confirm the Zung scales’ predictive ability against diagnoses made on the basis of a full clinical assessment. This research can also further explore whether more conservative clinical cut-offs should be applied, as suggested by these results. There may also be something to be gained by examining the differing natures of the ‘Misses’ and ‘False Positives’ produced by the differing scales. This may help identify areas to which one or the other scale is more suited. (The fact that the PHQ diagnoses are not definitive makes such an analysis inappropriate to the current study). For the time being, the current study lends support to the continuing use of the Zung scales, provided the errors regarding cut-off points that have plagued its use in the past be avoided.

## Abbreviations

DASS: Depression Anxiety Stress Scale
DSM: Diagnostic and Statistical Manual of Mental Disorders
MDD: Major Depressive Disorder
PHQ: Patient Health Questionnaire
PRIME-MD: Primary Care Evaluation of Mental Disorders
SAS: Zung Self Rating Anxiety Scale
SCID: Structured Clinical Interview for DSM Disorders
SDS: Zung Self Rating Depression Scale
*SS*^*ΔP*^: Semi-standardised coefficient

## References

[1] American Psychiatric Association (2013). Diagnostic and statistical manual of mental disorders.

[2] First MB, Gibbon M (2004). The structured clinical interview for DSM-IV Axis I disorders (SCID-I) and the structured clinical interview for DSM-IV Axis II disorders (SCID-II).

[3] Kupfer DJ (2005). Dimensional models for research and diagnosis: a current dilemma. J Abnorm Psychol.

[4] Trull TJ (2005). Clinical psychology.

[5] Andrews G, Henderson S, Hall W (2001). Prevalence, comorbidity, disability and service utilisation overview of the Australian National Mental Health Survey. Br J Psychiatry.

[6] Kessler RC, Aguilar-Gaxiola S, Alonso J, Chatterji S, Lee S, Ormel J (2009). The global burden of mental disorders: an update from the WHO world mental health (WMH) surveys. Epidemiol Psichiatr Soc.

[7] Clarke LA, Watson D, Becker J, Kleinman A (1991). Theoretical and empirical issues in differentiating depression from anxiety. Psychosocial aspects of mood disorders.

[8] Fischer EH, Goethe JW (1997). Measurement of depression and anxiety for hospitalized depressed patients. Psychiatr Serv.

[9] Feldman LA (1993). Distinguishing depression and anxiety in self-report: evidence from confirmatory factor analysis on nonclinical and clinical samples. J Consult Clin Psychol.

[10] Tanaka-Matsumi J, Kameoka VA (1986). Reliabilities and concurrent validities of popular self-report measures of depression, anxiety, and social desirability. J Consult Clin Psychol.

[11] Zung WWK (1965). A self-rating depression scale. Arch Gen Psychiatry.

[12] Zung WWK (1971). A rating instrument for anxiety disorders. Psychosomatics.

[13] Dunstan DA, Scott N. Assigning clinical significance and symptom severity using the Zung scales: Levels of misclassification arising from confusion between Index and Raw scores. Submitted for publication. 10.1155/2018/9250972 PMC5828114 29610683

[14] Lovibond PF, Lovibond SH (1995). The structure of negative emotional states: comparison of the depression anxiety stress scales (DASS) with the Beck depression and anxiety inventories. Behav Res Ther.

[15] Antony MM, Bieling PJ, Cox BJ, Enns MW, Swinson RP (1998). Psychometric properties of the 42-item and 21-item versions of the depression anxiety stress scales in clinical groups and a community sample. Psychol Assessment.

[16] Crawford JR, Henry JD (2003). The depression anxiety stress scales (DASS): normative data and latent structure in a large non-clinical sample. Br J Clin Psychol.

[17] Spitzer RL (1999). Kroenke K, Williams JBW, patient health Questionaire primary study group. Validation and utility of a self-report version of PRIME-MD: the PHQ primary care study. JAMA.

[18] Kroenke K, Spitzer RL, Williams JB (2001). The Phq-9: validity of a brief depression severity measure. J Gen Intern Med.

[19] Katon W, Roy-Byrne PP (1991). Mixed anxiety and depression. J Abnorm Psychol.

[20] Norton PJ (2007). Depression anxiety and stress scales (DASS-21): psychometric analysis across four racial groups. Anxiety Stress Coping.

[21] Zung WWK (1974). The measurement of affects: depression and anxiety. Mod Probl Pharmacopsychiatry.

[22] Zung WWK (1980). How normal is anxiety?.

[23] Zung WWK (1973). From art to science: the diagnosis and treatment of depression. Arch Gen Psychiatry.

[24] Deforge BR, Sobal J (1989). Self-report depression scales in the elderly: the relationship between the CES-D and Zung. Int J Psychiatry Med.

[25] Knight RG, Waal-Manning HJ, Spears GF (1983). Some norms and reliability data for the state-trait anxiety inventory and the Zung self-rating depression scale. Br J Clin Psychol.

[26] Carroll BJ, Fielding JM, Blashki TG (1973). Depression rating scales: a critical review. Arch Gen Psychiatry.

[27] American Psychiatric Association, Diagnostic and statistical manual of mental disorders 4th ed., text rev. Washington DC: American Psychiatric Association; 2000.

[28] Aldrich JH, Nelson FD (1984). Linear, probability, logit, and probit models.

[29] Kaufman RL (1996). Comparing effects in dichotomous logistic regression: a variety of standardized coefficients. Soc Sci Q.

[30] Kessler RC, Nelson CB, McGonagle KA, Liu J, Swartz M, Blazer DG (1996). Comorbidity of DSM-III—R major depressive disorder in the general population: results from the US National Comorbidity Survey. Brit J Psychiat.

[31] Micallef L, Rodgers P. eulerAPE: Drawing area-proportional 3-Venn Diagrams using ellipses. PLoS ONE. 2014; doi:10.1371/journal.pone.0101717. 10.1371/journal.pone.0101717 PMC4102485 25032825

